# Level and Predictors of Catastrophic Health Expenditure Among Recipients of Ambulatory Surgical Care Services at Kyabirwa Surgical Center, Uganda

**DOI:** 10.7759/cureus.98000

**Published:** 2025-11-28

**Authors:** Ronard Tusiime, Shafiga Babirye, Job Nanyiri, Joseph Okello Damoi, Arthur Emoru, Moses Bakaleke Binoga, Daniel Mukisa, Gloria Kwagala, Linda Zhang, Anna Turumanya Kalumuna

**Affiliations:** 1 Finance, Global Surgical Initiatives Inc, Kyabirwa Surgical Center, Jinja, UGA; 2 Social Work, Global Surgical Initiatives Inc, Kyabirwa Surgical Center, Jinja, UGA; 3 Research and Development, Global Surgical Initiatives Inc, Kyabirwa Surgical Center, Jinja, UGA; 4 Surgery, Global Surgical Initiatives Inc, Kyabirwa Surgical Center, Jinja, UGA; 5 Anesthesiology, Global Surgical Initiatives Inc, Kyabirwa Surgical Center, Jinja, UGA; 6 Research, Global Surgical Initiatives Inc, Kyabirwa Surgical Center, Jinja, UGA

**Keywords:** ambulatory surgery, catastrophic health expenditure, kyabirwa surgical center, recipients of ambulatory surgery, standalone ambulatory surgical centers

## Abstract

Background: Standalone ambulatory surgical centers are gradually being established globally to minimize the cost barrier to surgery access as, for every dollar spent on surgery in non-ambulatory facilities, standalone ambulatory surgical centers spend less. However, even at ambulatory surgical facilities, patients still have to pay for surgery, which may potentially put them at risk of catastrophic health expenditure (CHE), albeit with varying risk factors. Notwithstanding this, assessments of the prevalence of devastating health expenditure among recipients of ambulatory surgery from standalone facilities have been scarce, not only in Uganda but also globally.

Objective: To assess the level and predictors of CHE among recipients of ambulatory surgical care services at Kyabirwa Surgical Center (KSC), Jinja, Uganda.

Method: An analytical cross-sectional design was used, with patients sampled using systematic random sampling. Data were collected through structured interviews, medical record abstraction, and document reviews and analyzed in Statistical Product and Service Solutions (SPSS, version 26; IBM SPSS Statistics for Windows, Armonk, NY) using frequency, cross-tabulations, distributions, and a binomial logit model.

Results: The prevalence of CHE among recipients of ambulatory surgery at KSC was 1.1%. The odds of experiencing CHE were 94.5% less among patients who had received open surgery (aOR = 0.055 (95% CI = 0.004-0.726); p = 0.028). The odds of experiencing CHE were 93.7% less among patients who had had elective surgeries (aOR = 0.063 (95% CI = 0.004 - 0.886); p = 0.040) compared to those who received emergency surgery.

Conclusion: The prevalence of CHE among patients who received surgical care at KSC was negligible. Thus, only a small proportion of patients who undergo ambulatory surgery done at KSC experience CHE, implying that the center significantly contributes to the target VI of the Lancet Commission of Global Surgery. However, the prevalence of surgery-related CHE is not yet at the target set by the commission. CHE among patients at the center is predicted by the type of surgery (laparoscopic) and its nature (emergency).

## Introduction

Surgery is expensive [1] and costs as much as $ 7,800 in low- and middle-income countries, making it unaffordable for most people, especially in these regions. One of the steadily emerging solutions to the cost barrier of surgery is the setup of standalone ambulatory surgical centers, which have proven to provide both quality and affordable surgery [2]. For every dollar spent on surgery in a non-standalone surgical facility, standalone ones spend between 0.4 and 0.7 dollars [2], which is significantly lower. However, even in a standalone surgical facility, patients still have to pay for surgery, which may put them at risk of catastrophic health expenditure (CHE). CHE generally refers to a situation in which a given household spends more than 10% of its total annual expenditure/income or more than 40% of its non-food consumption on healthcare costs [3]. In the context of surgical care, CHE can turn out to be the greatest barrier to accessing crucial quality postoperative care, increasing risk for severe postoperative outcomes [4]. Even when care is finally sought following the delay caused by CHE, the household from which the patient hails faces an even more pronounced cost of care, accruing from prolonged stay in admission and more costly treatments for the incident complications [5].

At that point, households become at risk of healthcare-related impoverishment, contrary to target VI (protection against CHE) set by the Lancet Commission on Global Surgery (LCGS) [6]. Specifically, the prevalence of CHE should be 0.0% among surgery patients [7], especially in ambulatory surgery settings, whose reputation is built on being a provider of comparatively more affordable surgery. Therefore, the association of CHE with ambulatory surgery may lead to slow progress in the proliferation of ambulatory surgical facilities and the low uptake of ambulatory surgery in favor of inpatient surgery, possibly leading to the non-achievement of global surgery targets. Nonetheless, there has been no quantification of the global prevalence of CHE among patients who underwent ambulatory surgery from standalone facilities in low- and middle-income countries. Previously, Kebede et al. [6] noted that there was a critical lack of research in the areas of surgical financing and economics to guide policy decisions at the country and institutional levels, with the same being true for Uganda. Nonetheless, it has been reported to be highly prevalent, at rates ranging from 2% to 45% in most studies [8]. In sub-Saharan Africa, rates ranging from 8 to 39.9% have been reported. Kyabirwa Surgical Center (KSC), Jinja, Uganda, provides unprecedentedly subsidized ambulatory surgery; however, it has been noticed that a considerable number of patients still find it a challenge to clear their surgical care bills. However, its magnitude and predictors have never been assessed since the KSC was established.

## Materials and methods

Study design and area

This study adopted an analytical cross-sectional design, which allowed for the study of only a representative section of respondents from whom both exposure and outcome data could be collected and analyzed for associations [9]. The KSC is a standalone ambulatory surgical facility located in Budondo sub-county, eastern central Uganda, and Busoga sub-region [10]. The center provides a wide range of ambulatory care and surgical services, including diagnostic services, general surgery, laparoscopic surgery, and endoscopy. KSC has been providing such services for well over five years, at subsidized costs, all in a bid to provide quality and affordable surgical care [10].

Study population and eligibility

The study population comprised patients (children, adolescents, and adults) who received ambulatory surgical care services from the KSC between January 2022 and December 2023. This time frame was targeted because it was during that time that cases symbolic of possible CHE among patients manifested the most at the KSC. Additionally, for the validity and accurate assessment of CHE, an accurate recall of household expenditures was necessary. This required that all respondents sampled were those who had undergone surgeries as recently as two years before the study and could thus better recall expenditures within that period. However, if the patient was an adolescent (10-19 years old), a child (<12 years old), or even an adult (>18 years old) who was not the household head, their household heads were also included to validate the assessment of total household consumption.

The study included only patients who had fully cleared their healthcare bills at the KSC, given that it is only with full payment that CHE can be accurately computed at any threshold, since the actual amount spent on healthcare was known. The study excluded patients who, on their own admission, were not in a position to validate the total consumption of their households, or those whose household representatives were not in a position to recall and provide valid estimates of household consumption.

Sample size and sampling procedures

The number of patients included in this study as respondents was determined using the formula in the study of Daniel [11], which is appropriate when the population size targeted is less than 10,000, as is the case with patients targeted at the KSC. Between 2022 and 2023, the number of patients who underwent ambulatory surgery was known and reported to be 1008, making it less than 10,000. Therefore, the formula by Daniel [11] was used, and it is given by

 X*N / (X + N -1) 

where n is the required sample size; X is the maximum sample size at a probability of 50%, given by Kish Leslie as 384; and X is the population size.

n = 384 x 1008 / 384 + (1008 - 1)

n = 387072 / 384 + (1007)

n = 278 patients

The sampling process started with access to the electronic record system of KSC, from which records of patients who sought and received ambulatory surgical care services between January 2022 and December 2023 were identified. Each respondent was contacted via telephone, with the purpose of the call being to establish their availability for the study, their eligibility, and their accessibility. This process was done consecutively until all patients who met the inclusion criteria and were available for interview were identified. Those patients constituted the sampling frame, consequent to which systematic random sampling was used to select the required 278 patients from the list.

Systematic random sampling was used at this stage because the emergent sampling frame comprised 987 patients, a number that allowed for the use of random sampling, without risking non-obtainment of the sample size. The systematic random sampling process began with the calculation of a sampling interval, which was achieved by dividing the population size of patients by the sample size of the study.

The interval (skip) was observed on the previously developed list, and each patient was contacted via telephone to inform them that they had been selected. Their locations were established, and the principal investigator contacted them in their respective communities for interviews.

Data collection techniques

Structured interviews are the gold standard data collection method for cross-sectional studies; however, in addition to structured interviews, the study also used medical record abstraction, given that there was a need to obtain clinical data such as the type of surgery, its duration, diagnosis, type of anesthesia, and medication administered. Document reviews of patient payment history at the KSC were also conducted to determine each patient’s expenditure on ambulatory surgery at the center. However, while health expenditures can be validly obtained from document reviews, household expenditures must be obtained from self-reporting. Therefore, caution was taken to ensure that recall bias was as minimal as possible on the part of the respondents. Interviews were conducted to facilitate recall for each respondent when assessing household expenditures. Expenditure for a given month was primarily based on what had been spent over the past seven days (one week) and was multiplied by four to compensate for monthly expenditure. All the data collected during the structured interviews were captured using a structured questionnaire.

Measurement of catastrophic health expenditure

Several thresholds have been developed to assess CHE, ranging from 10%, 15%, 25%, and 40%; however, while they are all applicable [12], the 10% threshold is the most widely applied, especially in low- and middle-income countries. A threshold of 10% is the official indicator used to track progress in universal health coverage (financial protection), especially when both food and non-food expenditures are considered [13]. Therefore, this was adopted in the present study, given the presumed higher likelihood of recall bias if the patients were asked to provide previous expenditure on only food or non-food items. Catastrophic health expenditure was determined if a given household (from which a patient was hailed) spent more than 10% of their total annual household expenditure/consumption (food and non-food) on surgical care at the KSC. Out-of-pocket expenditure was assessed based on the amount of money spent on diagnostic tests, admission fees, medicines, consumables, and consultation fees [12]. To further prevent recall bias, the study assessed household expenditure over the most recent 31 days (a month). It is that expenditure that was then extrapolated across 12 months to represent annual expenditure.

Data analysis

The data obtained were auto-generated in Microsoft Excel (Microsoft® Corp., Redmond, WA) and imported into Statistical Product and Service Solutions (SPSS, version 25; IBM SPSS Statistics for Windows, Armonk, NY) for analysis. Descriptive statistics (frequency distributions) were first analyzed to establish the proportions (frequencies and valid percentages) of each variable. This stage of analysis enabled the establishment of the prevalence of catastrophic health expenditures, which was the first objective. For the second, third, and fourth objectives of the study, further descriptive analyses using cross-tabulations were performed between each independent variable and outcome. This was followed by the use of a binomial logit model to analyze the relationships between the independent and dependent variables in the bivariate analysis, since the magnitude of the outcome was less than 10%. This yielded p-values, confidence intervals, and crude odds ratios. Variables with p-values less than 0.2 were included in the multivariable analysis. The consideration of a threshold of 0.2 for screening variables that could be considered for multivariate analysis allowed for the inclusion of more potential drivers into the multivariate model than would have been included if the threshold had been left at 5% [14]. Thus, with the screening threshold set at 0.2 (20%), it was possible to identify that CHE predictors were insignificant at the bivariate level, at the 5% threshold [14]. At the multivariable level, each variable from the bivariate stage was adjusted for confounders in a single model. All variables that were found to have p-values less than 0.05 were considered to be predictors of CHE.

Ethical considerations

This study was approved by the Uganda Christian University Research Ethics Committee (UCU-REC) under the number UCUREC-2024-849 and by the Uganda National Council of Science and Technology under the number SS2900ES. Permission to conduct this study was obtained from the KSC Research Committee and Administration. Consent was obtained from each adult patient (Appendix A), and assent was obtained from those who were below 18 years of age but above eight years of age, with full consent obtained from their parents or guardians. All data obtained were kept confidential, and all records and completed questionnaires were stored in the principal investigator’s lockable cabinet. Similarly, all data were analyzed on the principal investigator’s personal computer, which was password-protected. The right to privacy for each respondent was respected, and all interviews were conducted between the interviewer and the respondent or their representatives only. Before any interviews were conducted, it was emphasized that participation and withdrawal from the study were voluntary. Respondents’ names were not recorded during data collection and processing.

## Results

Sociodemographic characteristics

Table 1 shows the sociodemographic characteristics of the patients who were interviewed in this study. The majority were female (142, 51.1%), almost half were aged 50 or older (135, 48.6%), and more than three-quarters were formally educated (243, 87.4%). The majority were educated to the postsecondary level (132, 54.3%). More than one-third were urban dwellers (122, 43.9%) in the Anglican faith (97, 34.9%). More than two-thirds of participants were employed (189, 68.0%) or married/cohabiting (188, 67.6%).

**Table 1:** Sociodemographic characteristics of the respondents

Variable	n(%)
Sex of respondent	
Female	142 (51.1)
Male	136 (48.9)
Age	
<30 years	38 (13.7)
31 - 50 years	105 (37.8)
> 50 years	135 (48.6)
Formally educated	
Yes	243 (87.4)
No	35 (12.6)
Level of education	
Primary	109 (44.9)
Secondary	2 (0.8)
Post secondary	132 (54.3)
Residence description	
Rural	100 (36.0)
Urban	122 (43.9)
Peri-urban	56 (20.1)
Currently employed	
Yes	189 (68.0)
No	89 (32.0)
Current marital status	
Married/cohabiting	188 (67.6)
Single	30 (10.8)
Separated/divorced	27 (9.7)
Widow/widower	33 (11.9)

Catastrophic health expenditure assessment

In Table 2, the findings from the assessment of out-of-pocket expenditure on ambulatory surgical care at KSC, and the household expenditure of each patient are presented. More than three-quarters (220, 79.1%) of the patients spent between $140 and $250 on ambulatory surgery at KSC. More than one-third (100, 36.0%) of patients spent more than $583 per month on household expenses. When CHE was computed, almost all respondents (271, 97.5%) spent between 0.0 and 4% of their households’ monthly expenditure on the surgical care that they received from the KSC.

**Table 2:** Catastrophic health expenditure assessment CHE: catastrophic health expenditure

Variable	n(%)
Amount of money spent on ambulatory surgery	
$29-$139	227 (81.6)
$140-$250	31 (11.2)
$251-$361	7 (2.5)
$362-$472	13 (4.7)
Total annual household consumption	
$29-$139	69 (24.8)
$140-$250	44 (15.8)
$251-$361	21 (7.6)
$362-$472	22 (7.9)
$473-$583	22 (7.9)
>$583	100 (36.0)
CHE range	
0.0-4%	271 (97.5)
5-9%	4 (1.4)
10% or more	3 (1.1)

Prevalence of catastrophic health expenditure

**Figure 1:**
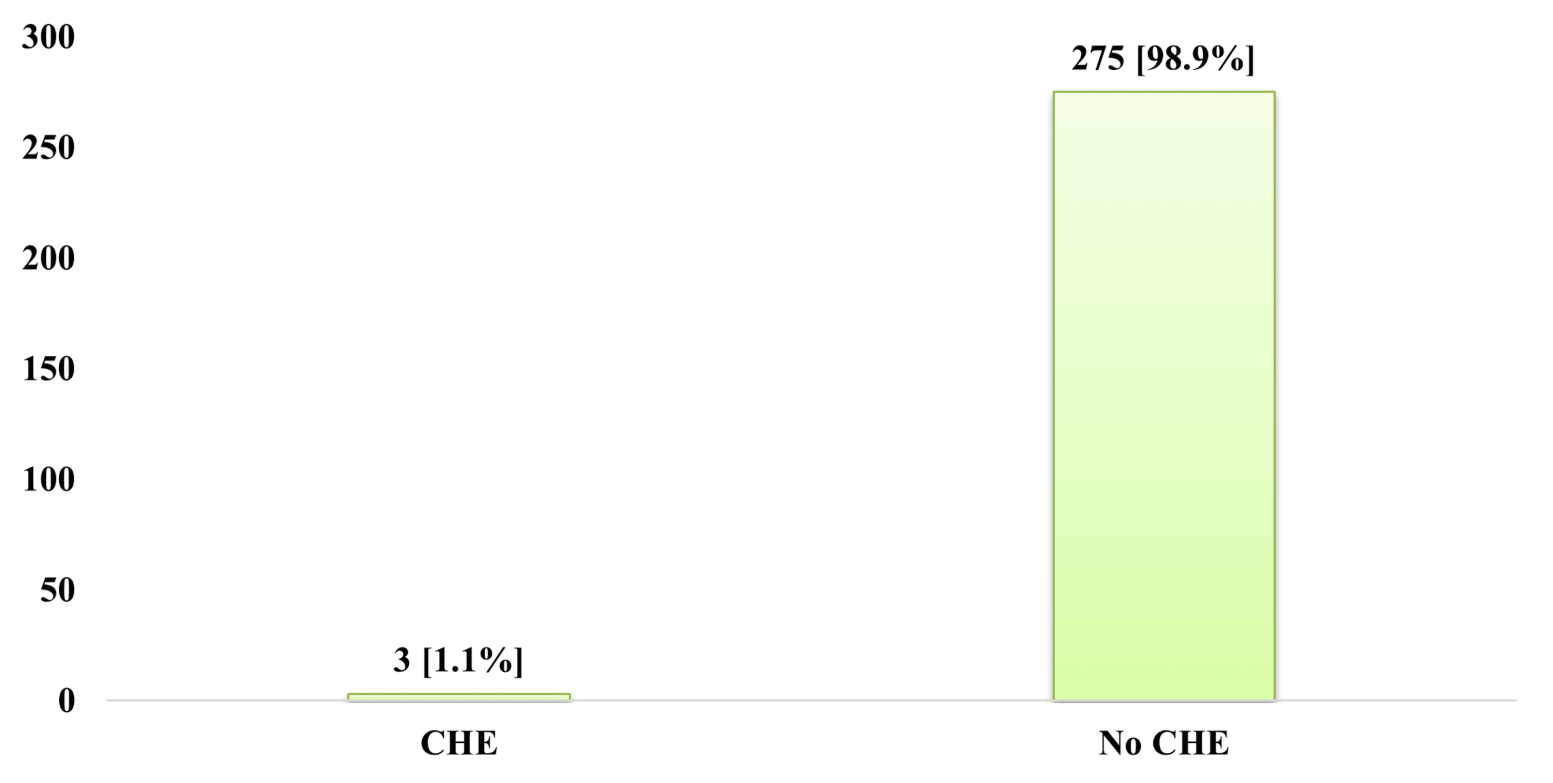
The prevalence of catastrophic health expenditure (CHE) among the recipients of ambulatory surgical care services at KSC KSC: Kyabirwa Surgical Center

Figure 1 shows the quantified prevalence of CHE among patients who received ambulatory surgery at KSC. Considering a threshold of 10%, the study established that the prevalence of catastrophic health expenditures among recipients of ambulatory surgical care services at the KSC was 1.1% (n = 3).

Predictors of catastrophic health expenditure

In Table 3, key findings from the analysis of CHE predictors are shown. Only two characteristics were found to have statistically significant relationships with CHE among patients who received surgical care at KSC. These included the type of surgery received and the nature of the surgery (emergency or elective). The odds of experiencing CHE were 94.5% lower among patients who underwent open surgery (aOR = 0.055 (95% CI = 0.004-0.726); p = 0.028) than among those who underwent laparoscopic surgery. The odds of experiencing CHE were 93.7% lower among patients who had undergone elective surgeries (aOR = 0.063 (95% CI = 0.004-0.886); p = 0.040) than those who had undergone emergency surgeries.

**Table 3:** Predictors of catastrophic health expenditure (CHE) among recipients of ambulatory surgical care services at KSC *Indicates a statistically significant p-value KSC: Kyabirwa Surgical Center

CHE status							
Variable		CHE	No CHE	cOR (95% CI)	P value	aOR (95% CI)	P value
Type of surgery received							
Open surgery	220 (85.9)	1 (0.5%)	219 (99.5%)	0.078 (0.007-0.880)	0.039	0.055 (0.004-0.726)	0.028*
Laparoscopic	36 (14.1)	2 (5.6%)	34 (94.4%)	1.000		1.000	
Nature of surgery received (emergency or elective)							
Elective	230 (82.7)	1 (0.4%)	229 (99.6%)	0.100 (0.009-1.131)	0.063	0.063 (0.004-0.886)	0.040*
Emergency	48 (17.3)	2 (4.2%)	46 (95.8%)	1.000		1.000	

## Discussion

According to the LCGS, the prevalence of CHE should be 0.0% among surgical patients. There is no doubt that ambulatory surgery will help achieve this target. This has been demonstrated in this study: only one in every 100 patients who received surgery from the KSC experienced CHE, which is perhaps one of the lowest prevalence of CHE ever recorded in a surgical context. The prevalence in this study was lower than 2.8% in Malaysia, 1.69% in Malaysia, 8.2% in Nigeria, 1.37% in Malawi, 3.94% in Togo, 22.5% in Ethiopia, 14.98% in Ethiopia, 17.3% in Ethiopia, and 5% in Ethiopia [15-18]. This comparison is a testament to the fact that the KSC lives up to its vision of providing affordable, high-quality surgical care to the rural population in Uganda, particularly eastern Uganda.

It is also a further testament to the fact that ambulatory surgery is comparatively cheaper than inpatient surgery, given that all the aforementioned studies were conducted in facilities that were not of the standalone ambulatory type or dedicated ambulatory surgery providing facilities. Very few studies have reported a surgery-related CHE prevalence lower than that in this study, and they included a study by Naidu et al. [19], who reported a CHE prevalence of 0.8%. The difference in findings arose from the fact that the study by Naidu et al. [19] was conducted among surgical patients in a public hospital in South Africa, where healthcare should ideally be free. Thus, compared with patients at Kyabirwa, those in the study by Naidu et al. [19] barely spent money out of pocket, implying that they were comparatively less likely to experience CHE. Nonetheless, despite the very low prevalence of CHE among patients who received surgical care from the KSC, the prevalence is still higher than the target of 0.0% set by the LCGS. Therefore, there is a need for interventions instituted by the KSC administration to reduce the apparent CHE incidence to 0%, which will be possible with evidence of what predicts CHE.

Two predictors of CHE were identified in this study: the type and nature of the surgery. This finding generally implies that in an ambulatory setting, such as the KSC, only the clinical characteristics of the patient, specifically the modalities of the surgery performed, matter when it comes to CHE risk. This finding was expected because both the type and nature of surgery determine the amount of money a patient must get out of pocket, even at the KSC. This study determined that the odds of experiencing CHE were 94.5% lower among patients who underwent open surgery compared to those who underwent laparoscopic surgery. This finding is inconsistent with those of previous studies [20-22,23], in which laparoscopic surgery was associated with a lower risk of CHE than open surgery. Some studies have reported that laparoscopic procedures are less expensive than open surgery [24], whereas others have found it to be more expensive [25]. In the context of KSC, laparoscopy is a procedure for complex major surgeries, such as cholecystectomy, and such laparoscopic procedures are the most expensive on the center's price list. Very few open surgical procedures in Kyabirwa cost as much as laparoscopic procedures, implying that even with subsidization, a patient who underwent laparoscopy in Kyabirwa paid 100% more than one who underwent open surgery, putting them at risk of CHE.

Regarding the nature of surgery, the study found that the odds of experiencing CHE were 93.7% lower among patients who had undergone elective surgery than among those who had undergone emergency surgery. This finding is consistent with the findings of Tefera et al. [26] and Obembe et al. [27] and was expected given that emergency surgeries are associated with more severe disease [28], which requires more complex surgeries, including laparoscopy, to be performed. Thus, based on the type of surgery required in emergencies, patients who need emergency surgery tend to spend more time out-of-pocket [29] than those who require elective surgeries. In addition to the high costs associated with the type of surgery, emergency surgeries, by virtue of being typified by the presence of severe disease, require more extensive postoperative patient monitoring [30], and hence, a greater number of days in admission. Such a prolonged stay on admission results in higher costs of care, given that each day on admission is charged [30], even in the context of KSC, which ideally ought to provide same-day surgery. Thus, it happens that patients who undergo emergency surgeries literally dig more out of their pockets than those who undergo elective surgeries since they honor their surgery appointments and show up for surgery in a comparatively better clinical state.

Limitations

While clinical and health expenditure data were collected from each patient's record, data on total household consumption were obtained solely from the patient's self-reports, suggesting that there may have been some room for exaggeration in the assessment of total household consumption. However, as a countermeasure, the interviewers endeavored to facilitate respondents’ recall of their household consumption based on a typical month or week, rather than a full year, since a year would have increased the risk of recall bias. The monthly consumption data obtained in this regard were multiplied by 12 to represent a typical year of expenditure for a given household. Another limitation of this study was the limited number of CHE events, which possibly made the regression model unstable, leading to the yield of inflated ratios. The study did not perform sensitivity analysis with alternative CHE thresholds and hence did not reveal whether the number of CHE events could change with a different threshold.

## Conclusions

The prevalence of CHE among patients who received surgical care at the KSC was negligible. Only a small proportion of patients who undergo ambulatory surgery at KSC experience CHE, implying that the center significantly contributes to the target VI of the Lancet Commission of Global Surgery, although the prevalence is not yet at the target set by the Lancet Commission of Global Surgery. Catastrophic health expenditure among patients in Kyabirwa is predicted by the type of surgery (laparoscopic) and its nature (emergency vs. elective surgery). Therefore, its total elimination can be achieved by strengthening community-based health education outreaches, in which a component of risk education ought to be included to encourage patients with surgical diseases to seek care before those conditions become severe. Laparoscopic procedures may be inevitable for some patients with specific surgically treatable diseases; therefore, the reduction in CHE risk among patients treated with the laparoscopic technique may require administrative intervention, as indicated by subsidies for patients whose economic assessment puts them in the category of those who require subsidized care.

## Appendices

Appendix A: Consent form

Title of the study: Level and Predictors of Catastrophic Health Expenditure Among Recipients of Ambulatory Surgical Care Services at Kyabirwa Surgical Center, Uganda

Introduction: One of the greatest barriers to accessing surgical care, especially in low- and middle-income countries, is the high cost that comes with such care. However, while that barrier still exists for millions of people, there have been interventions like ambulatory surgery that have been put in place in order to minimize it. The concern is apparent; nonetheless, the possibility is that while ambulatory surgery, particularly that being provided at Kyabirwa Surgical Center, is heavily subsidized, there are several patients who may be experiencing catastrophic health expenditure.

Objective: To assess the level and predictors of Catastrophic Health Expenditure among recipients of ambulatory surgical care services at Kyabirwa Surgical Center

Target population: The study will target patients who received ambulatory services (medical or surgical) from Kyabirwa Surgical Center

Study population: The actual study population will be patients (children, adolescents, and adults) who will have received ambulatory surgical care services from Kyabirwa Surgical Center, between the years 2022 and 2023

Why you have been chosen: You have been chosen to be one of the participants in this study because you received ambulatory care from Kyabirwa Surgical Center between the years 2022 and 2023

Study procedures: This study is interested in collecting quantifiable data, and so, participants will be asked several closed-ended questions (those with predetermined response options) for about 30 minutes. So, we do not expect any fatigue on your part. We shall ask you questions to do with your socio-demographic characteristics, clinical characteristics, and intra-household ones. That will be in addition to catastrophic health expenditure, for which we will ask you to recall your typical monthly expenditure in the year during which you sought ambulatory care.

Risks and benefits of your participation: You can be assured that you will not be harmed in any way as a respondent in this study, because your role will only involve responding to some closed-ended questions and not participating in any interventions.  The findings of this study will be of significant benefit to you as an individual and to others who may seek ambulatory surgery from Kyabirwa Surgical Center. With your responses, Kyabirwa Surgical Center will be able to know the proportion of its patients who face CHE risk, who they are, and how to protect them from such expenditure.

Confidentiality and anonymity: When you choose to participate in this study, you can be assured that all the information you will provide will be handled with confidentiality. Your names will neither be captured on this consent form nor on the questionnaire to which you will respond. You will only be requested to append a signature on the questionnaire, onto which you will respond. We will not share the information you will provide to any of the local leaders in this area, or the authorities at this health facility you are attached to, but if we are to share it, then your identity will be concealed.

Privacy: If you decide to be a participant, the interviews you will be engaged in will be conducted in private; only you and the interviewer will be able to listen in on each other’s conversation without interference from any third parties.

Voluntary participation and withdrawal: Your participation in this study is purely voluntary; it is upon your discretion to participate or not to. There will be no repercussions if you decide not to participate in this study; you will still be in a position to access all the healthcare you need from this hospital, as you have been.

Incentives: Since your participation in this study is voluntary, you will not be paid for your participation. However, compensation for your time as a study participant will be arranged, to the tune of 10,000/-

Contacts: In case of any inquiries about the study, please feel free to contact the principal investigator on Tel:  074 860269

Consent form

Name of Researcher: Babirye Shafiga

Please initial all boxes

1. I confirm that I have read and understand the information sheet dated ………………… for the above study. I have had the opportunity to consider the information, ask questions, and have had these answered satisfactorily.

2. I understand that my participation is voluntary and that I am free to withdraw at any time without giving any reason, without my medical care or legal rights being affected.

3.  I understand that relevant sections of my medical notes and data collected during the study may be looked at by the interviewers and individuals from SHU, where it is relevant to my taking part in this research.  I permit these individuals to have access to my records, although anonymously.

4. I agree to take part in the above study.

Name of Participant                            Date                                         Signature

Name of Person                                  Date                                         Signature

## References

[1] Aboutorabi A, Radinmanesh M, Rezapour A, Afshari M, Taheri G (2020). A comparison of global surgery tariffs and the actual cost of bills at Hazrate Rasoole Akram Educational and Medical Center. Cost Eff Resour Alloc.

[2] Federico VP, Nie JW, Butler A, Phillips F (2023). Medicare procedural costs in ambulatory surgery centers versus hospital outpatient departments for spine surgeries. Spine J.

[3] (2025). Households with out-of-pocket payments greater than 40% of capacity to pay for health care (food, housing and utilities approach - developed by WHO/Europe). https://www.who.int/data/gho/indicator-metadata-registry/imr-details/4989.

[4] Bolmers MD, de Jonge J, Bom WJ (2022). In-hospital delay of appendectomy in acute, complicated appendicitis. J Gastrointest Surg.

[5] Dharap SB, Barbaniya P, Navgale S (2022). Incidence and risk factors of postoperative complications in general surgery patients. Cureus.

[6] Kebede MA, Tor DS, Aklilu T (2023). Identifying critical gaps in research to advance global surgery by 2030: a systematic mapping review. BMC Health Serv Res.

[7] Zadey S, Iyer H, Nayan A (2023). Evaluating the status of the Lancet Commission on Global Surgery indicators for India. Lancet Reg Health Southeast Asia.

[8] Zhang F, Jiang J, Yang M, Zou K, Chen D (2023). Catastrophic health expenditure, incidence, trend and socioeconomic risk factors in China: a systematic review and meta-analysis. Front Public Health.

[9] Savitz DA, Wellenius GA (2023). Can cross-sectional studies contribute to causal inference? It depends. Am J Epidemiol.

[10] (2025). Kyabirwa Surgical Center. https://www.kyabirwasc.org/.

[11] Daniel WW (1999). Biostatistics: A Foundation for Analysis in the Health Sciences, 7th Edition. Biostatistics: A Foundation for Analysis in the Health Sciences.

[12] Mulaga AN, Kamndaya MS, Masangwi SJ (2021). Examining the incidence of catastrophic health expenditures and its determinants using multilevel logistic regression in Malawi. PLoS One.

[13] Wagstaff A, Flores G, Hsu J (2018). Progress on catastrophic health spending in 133 countries: a retrospective observational study. Lancet Glob Health.

[14] Bendel RB, Afifi AA (1977). Comparison of stopping rules in forward “stepwise” regression. J Am Stat Assoc.

[15] Sayuti M, Sukeri S (2022). Assessing progress towards Sustainable Development Goal 3.8.2 and determinants of catastrophic health expenditures in Malaysia. PLoS One.

[16] Hussein N, Ng CW, Ramli R (2024). Assessing catastrophic health expenditure and impoverishment in adult asthma care: a cross-sectional study of patients attending six public health clinics in Klang district, Malaysia. BMC Health Serv Res.

[17] Okedo-Alex IN, Akamike IC, Ezeanosike OB, Uneke CJ (2019). A review of the incidence and determinants of catastrophic health expenditure in Nigeria: implications for universal health coverage. Int J Health Plann Manage.

[18] Getachew N, Shigut H, Jeldu Edessa G, Yesuf EA (2023). Catastrophic health expenditure and associated factors among households of non community based health insurance districts, Ilubabor zone, Oromia regional state, southwest Ethiopia. Int J Equity Health.

[19] Naidu P, Ataguba JE, Shrime M, Alkire BC, Chu KM (2022). Surgical catastrophic health expenditure and risk factors for out-of-pocket expenditure at a South African public sector hospital. World J Surg.

[20] Zadey S, Mueller J, Fitzgerald TN (2022). Improving access to laparoscopic surgery in low- and middle-income countries. JAMA Surg.

[21] Bryce-Alberti M, Campos LN, Dey T (2023). Availability of laparoscopic surgery in Mexico's public health system: a nationwide retrospective analysis. Lancet Reg Health Am.

[22] Mishra A, Bains L, Jesudin G, Aruparayil N, Singh R, Shashi Shashi (2020). Evaluation of gasless laparoscopy as a tool for minimal access surgery in low-to middle-income countries: a phase II noninferiority randomized controlled study. J Am Coll Surg.

[23] Genetu A, Gezahegn D, Getachew H, Deneke A, Bekele A (2022). Financial risk of emergency abdominal surgery: a cross sectional study from Ethiopia. BMC Health Serv Res.

[24] Chok AY, Tan IE, Zhao Y (2023). Clinical outcomes and cost comparison of laparoscopic versus open surgery in elderly colorectal cancer patients over 80 years. Int J Colorectal Dis.

[25] Gehrman J, Björholt I, Angenete E, Andersson J, Bonjer J, Haglind E (2017). Health economic analysis of costs of laparoscopic and open surgery for rectal cancer within a randomized trial (COLOR II). Surg Endosc.

[26] Tefera GM, Feyisa BB, Umeta GT, Kebede TM (2020). Predictors of prolonged length of hospital stay and in-hospital mortality among adult patients admitted at the surgical ward of Jimma University Medical Center, Ethiopia: prospective observational study. J Pharm Policy Pract.

[27] Obembe TA, Levin J, Fonn S (2021). Prevalence and factors associated with catastrophic health expenditure among slum and non-slum dwellers undergoing emergency surgery in a metropolitan area of south western Nigeria. PLoS One.

[28] Yap A, Olatunji BT, Negash S (2023). Out-of-pocket costs and catastrophic healthcare expenditure for families of children requiring surgery in sub-Saharan Africa. Surgery.

[29] Mukherjee R, Samanta S (2019). Surgical emergencies in pregnancy in the era of modern diagnostics and treatment. Taiwan J Obstet Gynecol.

[30] Follette CJ, Grimes AD, Detelich DM, Martin RS (2024). Finding value in emergency general surgery. Curr Surg Rep.

